# Driving Risk Assessment and Advice Provision for Inpatients Based on Features of Illness, Treatment and Driver and Vehicle Licensing Agency (DVLA) Guidelines

**DOI:** 10.1192/bjo.2022.473

**Published:** 2022-06-20

**Authors:** Oghenefejiro Ofovwe, Nduka Nzekwue

**Affiliations:** Kent and Medway NHS and Social Care Partnership Trust, Kent, United Kingdom

## Abstract

**Aims:**

The aim of the project was to improve the routine incorporation of driving advice based on Driver and Vehicle Licensing Agency (DVLA) guidance into discharge planning by responsible inpatient teams. This would optimize patient safety, demonstrate good clinical practice (trust and professional body values) and minimize/prevent the emergence of accidents/unfair loss of licenses/unfair attribution of driving accidents caused by people who have been under recent or ongoing inpatient care.

**Methods:**

The following questions: “Do you have a valid license”, “Do you own/have access to a vehicle”, “Do you currently drive” were developed as a standard template for gathering patients’ driving information.

These questions were embedded within:

1. Barriers to ward discharge discussions
2. Trust-wide communications via screensaver and circular

Answers to these questions were to be clearly documented on patient's records to serve as prompts for the responsible discharging team to take up providing the appropriate advice.

After a specified period, the electronic discharge notification (EDN) database was searched for patients with relevant diagnosis who were discharged from all the general adult/older adult acute inpatient wards within a specified period. The patients’ records were then checked for documentation of relevant driving information evidenced by documentation of answers to the screening questions as well as recorded evidence of DVLA discussion/advice held since date of diagnosis or admission.

The standards audited against were all patients:

1. should have their driving licence status recorded during their admission
2. should have their access to a vehicle recorded during their admission
3. with a relevant mental health diagnosis should have a record of advice regarding driving given in bespoke and DVLA informed manner during ward discharge planning by the responsible discharging team
4. should have documentation of the outcome of the driving advice given by the responsible team in their records

**Results:**

28 patients with relevant DVLA notifiable mental health conditions were audited. 11% (n = 3) had driving licence status recorded. 14% (n = 4) had access to a vehicle recorded. 7% (n = 2) had driving advice given. Only one patient had outcome of driving advice recorded. No best practice was identified.

**Conclusion:**

Documentation of driving information, DVLA signposting advice and outcome for patients with relevant mental health diagnosis is a crucial part of patient risk assessment and management as these patients are not free from posing a driving risk on discharge. The trust is implementing actions to improve the routine incorporation of driving advice based on DVLA guidance into discharge planning.

